# Supplementary material for: Highly Conserved Elements and Chromosome Structure Evolution in Mitochondrial Genomes in Ciliates
Source: Life (Basel). 2017 Feb 27;7(1):9. doi: 10.3390/life7010009 (PMC5370409; doi:10.3390/life7010009)
Supplement: Supplementary file 2 [file life-07-00009-s001.zip › AddFile2.pdf]

Reconstruction of mitochondrial chromosome structures in ciliates

The root and its two direct descendants have two chromosomes (linear and circular). Other internal nodes have one linear chromosome. Each non-terminal node is represented by its two most distant descendant leafs. Events are shown for the incoming tree edge.

Node	Chromosome Structure	Events
***T. thermophila* (*l*)**	*trnY *rnl_a_1 *trnL_1 *rnl_b_1 *ymf57 *ymf66 *ymf76 *rps13 *rps3 *rps19 *rpl2 *ymf74 *nad10 *rps12 *nad2 nad7 rps14 ymf60 *ymf64 *ymf75 *trnF *nad1_b *atp9 *ymf63 *ymf65 *ymf69 *ymf59 *rpl16 *yejR *ymf61 *trnH *nad3 *ymf72 *nad4L *nad9_2 *nad9_1 *ymf77 cob nad5 cox2 rns_a rns_b ymf56 ymf67 trnW ymf68 ymf71 cox1 nad1_a ymf62 rpl14 trnE ymf70 nad4 ymf73 rnl_b_2 trnL_2 rnl_a_2 trnM (L)	no events
***T. malaccensis* (*l*)**	*trnY *rnl_a_1 *trnL_1 *rnl_b_1 *ymf57 *ymf66 *ymf76 *rps13 *rps3 *rps19 *rpl2 *ymf74 *nad10 *rps12 *nad2 nad7 rps14 ymf60 *ymf64 *ymf75 *trnF *nad1_b *atp9 *ymf63 *ymf65 *ymf69 *ymf59 *rpl16 *yejR *ymf61 *trnH *nad3 *ymf72 *nad4L *nad9_2 *nad9_1 *ymf77 cob nad5 cox2 rns_a rns_b ymf56 ymf67 trnW ymf68 ymf71 cox1 nad1_a ymf62 rpl14 trnE ymf70 nad4 ymf73 rnl_b_2 trnL_2 rnl_a_2 trnM (L)	no events
T. thermophila* – *T. malaccensis	*trnY *rnl_a_1 *trnL_1 *rnl_b_1 *ymf57 *ymf66 *ymf76 *rps13 *rps3 *rps19 *rpl2 *ymf74 *nad10 *rps12 *nad2 nad7 rps14 ymf60 *ymf64 *ymf75 *trnF *nad1_b *atp9 *ymf63 *ymf65 *ymf69 *ymf59 *rpl16 *yejR *ymf61 *trnH *nad3 *ymf72 *nad4L *nad9_2 *nad9_1 *ymf77 cob nad5 cox2 rns_a rns_b ymf56 ymf67 trnW ymf68 ymf71 cox1 nad1_a ymf62 rpl14 trnE ymf70 nad4 ymf73 rnl_b_2 trnL_2 rnl_a_2 trnM (L)	1 duplication
***T. paravorax* (*l*)**	*trnY *rnl_a_1 *trnL_1 *rnl_b_1 *ymf57 *ymf66 *ymf76 *rps13 *rps3 *rps19 *rpl2 *ymf74 *nad10 *rps12 *nad2 nad7 rps14 ymf60 *ymf64 *ymf75 *trnF *nad1_b *atp9 *ymf63 *ymf65 *ymf69 *ymf59 *rpl16 *yejR *ymf61 *trnH *nad3 *ymf72 *nad4L *nad9_1 *ymf77 cob nad5 cox2 rns_a rns_b ymf56 ymf67 trnW ymf68 ymf71 cox1 nad1_a ymf62 rpl14 trnE ymf70 nad4 ymf73 rnl_b_2 trnL_2 rnl_a_2 trnM (L)	no events
T. paravorax* – *T. malaccensis	*trnY *rnl_a_1 *trnL_1 *rnl_b_1 *ymf57 *ymf66 *ymf76 *rps13 *rps3 *rps19 *rpl2 *ymf74 *nad10 *rps12 *nad2 nad7 rps14 ymf60 *ymf64 *ymf75 *trnF *nad1_b *atp9 *ymf63 *ymf65 *ymf69 *ymf59 *rpl16 *yejR *ymf61 *trnH *nad3 *ymf72 *nad4L *nad9_1 *ymf77 cob nad5 cox2 rns_a rns_b ymf56 ymf67 trnW ymf68 ymf71 cox1 nad1_a ymf62 rpl14 trnE ymf70 nad4 ymf73 rnl_b_2 trnL_2 rnl_a_2 trnM (L)	no events

Node	Chromosome Structure	Events
***T. pigmentosa* (*l*)**	*trnY *rnl_a_1 *trnL_1 *rnl_b_1 *ymf57 *ymf66 *ymf76 *rps13 *rps3 *rps19 *rpl2 *ymf74 *nad10 *rps12 *nad2 nad7 rps14 ymf60 *ymf64 *ymf75 trnF *nad1_b *atp9 *ymf63 *ymf65 *ymf69 *ymf59 *rpl16 *yejR *ymf61 trnH *nad3 *ymf72 *nad4L *nad9_1 *ymf77 cob nad5 cox2 rns_a rns_b ymf56 ymf67 trnW ymf68 ymf71 cox1 nad1_a ymf62 rpl14 trnE ymf70 nad4 ymf73 rnl_b_2 trnL_2 rnl_a_2 trnM (L)	2 inversions
T. paravorax* –<br>*T. pigmentosa	*trnY *rnl_a_1 *trnL_1 *rnl_b_1 *ymf57 *ymf66 *ymf76 *rps13 *rps3 *rps19 *rpl2 *ymf74 *nad10 *rps12 *nad2 nad7 rps14 ymf60 *ymf64 *ymf75 *trnF *nad1_b *atp9 *ymf63 *ymf65 *ymf69 *ymf59 *rpl16 *yejR *ymf61 *trnH *nad3 *ymf72 *nad4L *nad9_1 *ymf77 cob nad5 cox2 rns_a rns_b ymf56 ymf67 trnW ymf68 ymf71 cox1 nad1_a ymf62 rpl14 trnE ymf70 nad4 ymf73 rnl_b_2 trnL_2 rnl_a_2 trnM (L)	no events
***T. pyriformis* (*l*)**	*trnY *rnl_a_1 *trnL_1 *rnl_b_1 *ymf57 *ymf66 *ymf76 *rps13 *rps3 *rps19 *rpl2 *ymf74 *nad10 *rps12 *nad2 nad7 rps14 rp16 *ymf64 *ymf75 *trnF *nad1_b *atp9 *ymf63 *ymf65 *ymf69 *ymf59 *rpl16 *yejR *ymf61 *trnH *nad3 *ymf72 *nad4L *nad9_1 *ymf77 cob nad5 cox2 rns_a rns_b ymf56 ymf67 trnW ymf68 ymf71 cox1 nad1_a ymf62 rpl14 trnE ymf70 nad4 ymf73 rnl_b_2 trnL_2 rnl_a_2 trnM (L)	1 gene substitution
T. paravorax* –<br>*T. pyriformis	*trnY *rnl_a_1 *trnL_1 *rnl_b_1 *ymf57 *ymf66 *ymf76 *rps13 *rps3 *rps19 *rpl2 *ymf74 *nad10 *rps12 *nad2 nad7 rps14 ymf60 *ymf64 *ymf75 *trnF *nad1_b *atp9 *ymf63 *ymf65 *ymf69 *ymf59 *rpl16 *yejR *ymf61 *trnH *nad3 *ymf72 *nad4L *nad9_1 *ymf77 cob nad5 cox2 rns_a rns_b ymf56 ymf67 trnW ymf68 ymf71 cox1 nad1_a ymf62 rpl14 trnE ymf70 nad4 ymf73 rnl_b_2 trnL_2 rnl_a_2 trnM (L)	6 gene gains
***I. multifiliis* (*l*)**	*trnY *rnl_a_1 *rnl_b_1 *ymf66 *ymf57 *ymf76 *rps13 *rps3 *rps19 *rpl2 *nad10 *rps12 *nad2 nad7 rps14 ymf60 *ymf64 *ymf75 *trnF *atp9 *ymf63 *ymf65 *ymf59 *rpl16 *yejR *ymf61 *nad3 *nad4L *nad9_1 *ymf77 *nad1_b cob nad5 cox2 rns_a rns_b ymf67 trnW ymf68 cox1 nad1_a ymf62 rpl14 ymf70 nad4 ymf73 trnE rnl_b_2 rnl_a_2 trnY_2 (C)	2 gene losses,<br>1 gene substitution,<br>2 transpositions,<br>1 chromosome cycling
I. multifiliis* –<br>*T. Pyriformis	*trnY *rnl_a_1 *rnl_b_1 *ymf57 *ymf66 *ymf76 *rps13 *rps3 *rps19 *rpl2 *nad10 *rps12 *nad2 nad7 rps14 ymf60 *ymf64 *ymf75 *trnF *nad1_b *atp9 *ymf63 *ymf65 *ymf59 *rpl16 *yejR *ymf61 *trnH *nad3 *nad4L *nad9_1 *ymf77 cob nad5 cox2 rns_a rns_b ymf56 ymf67 trnW ymf68 cox1 nad1_a ymf62 rpl14 trnE ymf70 nad4 ymf73 rnl_b_2 rnl_a_2 trnM (L)	1 region insertion,<br>4 gene gains,<br>1 gene substitution,<br>1 transversion,<br>1 transposition,<br>1 insertion of circular chromosome into linear one

Node	Chromosome Structure	Events
***P. aurelia* (*l*)**	*trnY *rnl_b *trnM *rnl_a *ymf66_1 *ymf66 *ymf57 *nad4 *ymf80 *rpl14 *ymf62 *nad1_a *cox1 *ymf68 *trnW *ymf67_b *ymf67_a *ymf56 *rns_b *rns_a *cox2 *nad5 *cob *ymf81 *ymf85 *rps13 *rps3 *rpl2 *ymf84 *nad10 *rps12 *nad2_a nad7 rps14 ymf79 ymf60 *ymf64 *ymf86 *ymf83 *atp9 *ymf63 *nad1_b *trnF *ymf65 *ymf65_1 *ymf78 *ymf59 *rpl16 *ymf82 *yejR *ymf61 *nad3 *nad4L *nad9_1 (L)	2 gene gains<br>3 duplications,<br>2 gene losses,<br>1 region insertion
***P. caudatum* (*l*)**	*trnY *rnl_a_1 *rnl_b_1 *ymf66 *ymf57 *nad4 *ymf80 *rpl14 *nad6 *nad1_a *cox1 *ymf68 *trnW *ymf67_a *ymf56 *rns_b *rns_a *cox2 *nad5 *cob *ymf76 *rps13 *rps3 *rps19 *rpl2 *ymf84 *nad10 *rps12 *nad2_a nad7 rps14 ymf79 rpl6 *ymf64 *ymf83 *atp9 *ymf63 *ymf87 *nad1_b *trnF *ymf65 *ymf78 *ymf59 *rpl16 *yejR *ymf61 *nad3 *nad4L *nad9_1 (L)	2 gene substitutions,<br>1 gene loss,<br>1 gene gain
P. aurelia – P. caudatum	*trnY *rnl_a_1 *rnl_b_1 *trnM *ymf66 *ymf57 *nad4 *ymf80 *rpl14 *ymf62 *nad1_a *cox1 *ymf68 *trnW *ymf67_a *ymf56 *rns_b *rns_a *cox2 *nad5 *cob *ymf76 *rps13 *rps3 *rps19 *rpl2 *ymf84 *nad10 *rps12 *nad2_a nad7 rps14 ymf79 ymf60 *ymf64 *ymf83 *atp9 *ymf63 *nad1_b *trnF *ymf65 *ymf78 *ymf59 *rpl16 *yejR *ymf61 *nad3 *nad4L *nad9_1 (L)	1 gene loss,<br>5 gene gains,<br>1 gene substitution,<br>1 inversion,<br>1 transposition,<br>1 transversion,<br>1 insertion of circular chromosome into linear one
P. aurelia – T. pyriformis	*trnM *ymf66 *ymf76 *rps13 *rps3 *rps19 *rpl2 *nad10 *rps12 *nad2_a nad7 rps14 ymf60 *ymf64 *trnF *ymf65 *ymf59 *rpl16 *yejR *ymf61 *trnH *nad3 *nad4L *nad9_1 cob nad5 cox2 rns_a rns_b ymf56 trnW ymf68 cox1 nad1_a ymf62 rpl14 trnE nad4 ymf57 rnl_b_1 rnl_a_1 trnY (L)<br>nad1_b ymf63 atp9 (C)	1 gene gain,<br>1 region insertion,<br>2 inversions,<br>4 transpositions,<br>2 transversions
***M. minuta* (*l*)**	*nad5 *ccmF *cytb *trnM *rnl *cox2 *rpl14 *cox1 *nad4L *rps3_b *trnW *rns *trnY trnF nad9_1 nad2_a rpl16 nad4 rps12 nad10 rpl2 rps4 nad7 nad1_b trnG atp9 nad3 trnH nad1_a (L)	no events
***M. crassus* (*l*)**	*nad5 *ccmF *cytb *trnM *rnl *cox2 *rpl14 *cox1 *nad4L *rps3_b *rns *trnY trnF nad9_1 nad2_a rpl16 nad4 rps12 nad10 rpl2 rps4 nad7 (L)	1 region deletion,<br>1 gene loss
M. minuta – M. crassus	*nad5 *ccmF *cytb *trnM *rnl *cox2 *rpl14 *cox1 *nad4L *rps3_b *trnW *rns *trnY trnF nad9_1 nad2_a rpl16 nad4 rps12 nad10 rpl2 rps4 nad7 nad1_b trnG atp9 nad3 trnH nad1_a (L)	3 gene losses,<br>1 region deletion,<br>3 gene gains,<br>1 inversion
***O. trifallax* (*l*)**	*rps2_sin *nad5 *nad5_iii_sin *nad5_ii_sin *nad5_i_sin *ccmF *nad1_a *trnH *cob *nad3 *rpl6_ii_sin *rpl6_i_sin *rps7 *rps3_a_sin *atp9 *rps8_sin2 *trnG *nad1_b *rps14 *nad7 *rps4 *rps13 *rps19 *rpl2 *nad10 *rps12 *nad4_i_sin *rpl16 *nad2_a_sin *nad2_b *trnL *rps10 *trnE *nad9 *nad9_i_sin *trnF trnY trnW rps3_b nad4L cox1 nad6_sin rpl14 cox2 trnM (L)	4 gene gains,<br>3 region insertions,<br>3 gene substitutions,<br>2 duplications

Node	Chromosome Structure	Events
M. minuta* – *O. trifallax	*nad5 *ccmF *nad1_a *trnH *cob *nad3 *atp9 *trnG *nad1_b *rps14 *nad7 *rps4 *rps13 *rps19 *rpl2 *nad10 *rps12 *nad4 *rpl16 *nad2_a *trnE *nad9_1 *trnF trnY trnW rps3_b nad4L cox1 rpl14 cox2 trnM (L)	4 gene gains,<br>1 inversion,<br>1 transposition,<br>2 transversions,<br>1 insertion of circular chromosome into linear one
***N. ovalis* (*l*)**	nad1_b nad1_a nad3 nad9_1 nad4 nad10 nad4L rpl14 nad2_a nad5 rpl2a_sin rpl2 nad7 rps14_sin rps8_sin1 rps4_sin rpl6 rps12 (L)	5 gene losses,<br>1 region insertion,<br>1 duplication,<br>1 gene substitution,<br>4 transpositions,<br>1 insertion of circular chromosome into linear one
M. minuta* – *N. ovalis	nad1_b atp9 rpl6 rps12 nad10 rpl2 rps19 rps13 nad7 rps14 *trnW *trnY trnF nad9_1 trnE nad2_a nad5 nad4L cox1 rpl14 cox2 trnM nad4 (L)<br>trnH nad1_a nad3 cob(C)	4 region deletion,<br>3 gene losses,<br>1 inversion,<br>3 transpositions,<br>2 transversions,<br>1 insertion of circular chromosome into linear one,<br>1 deletion of circular chromosome from linear one
**The tree root**	*cob *nad1_a *yejR *ymf61 *trnH *nad3 *nad4L *rpl14 *ymf62 *cox1 *ymf68 *trnW *ymf56 *rns_b *rns_a *ymf66 *rps13 *rps3 *rps19 *rpl2 *nad10 *rps12 nad9_1 trnE nad2_a nad5 cox2 trnM rpl16 ymf59 ymf65 nad7 rps14 nad4 ymf57 ymf60 *ymf64 *trnF trnY (L)<br>nad1_b ymf63 atp9 (C)	–
